# Flow cytometry may allow microscope-independent detection of holocentric chromosomes in plants

**DOI:** 10.1038/srep27161

**Published:** 2016-06-03

**Authors:** František Zedek, Pavel Veselý, Lucie Horová, Petr Bureš

**Affiliations:** 1 Department of Botany and Zoology, Masaryk University, Kotlarska 2, 611 37 Brno, Czech Republic

## Abstract

Two chromosomal structures, known as monocentric and holocentric chromosomes, have evolved in eukaryotes. Acentric fragments of monocentric chromosomes are unequally distributed to daughter cells and/or lost, while holocentric fragments are inherited normally. In monocentric species, unequal distribution should generate chimeras of cells with different nuclear DNA content. We investigated whether such differences in monocentric species are detectable by flow cytometry (FCM) as (i) a decreased nuclear DNA content and (ii) an increased coefficient of variance (CV) of the G1 peak after gamma radiation-induced fragmentation. We compared 13 monocentric and 9 holocentric plant species. Unexpectedly, monocentrics and holocentrics did not differ with respect to parameters (i) and (ii) in their response to gamma irradiation. However, we found that the proportion of G2 nuclei was highly elevated in monocentrics after irradiation, while holocentrics were negligibly affected. Therefore, we hypothesize that DNA-damaging agents induce cell cycle arrest leading to endopolyploidy only in monocentric and not (or to much lesser extent) in holocentric plants. While current microscope-dependent methods for holocentrism detection are unreliable for small and numerous chromosomes, which are common in holocentrics, FCM can use somatic nuclei. Thus, FCM may be a rapid and reliable method of high-throughput screening for holocentric candidates across plant phylogeny.

Eukaryotic chromosomes are nucleoprotein vehicles that mediate the transmission of genetic material to daughter cells by attaching to spindle microtubules during cell division. Chromosomes are typically represented as sausage-like structures with a constriction at the centromere. The centromere is the region where the kinetochore, which interacts with spindle microtubules, is formed. However, so-called monocentric chromosomes are only one of the 2 types of eukaryotic chromosomes. Various plant and animal lineages have independently evolved holocentric chromosomes, which lack centromeres; instead, they assemble their kinetochore along their entire length^1^,^2^.

Owing to their extended kinetochore and chromatid cohesion, holocentric chromosomes are prone to chromosomal fission and fusion events^3^,^4^,^5^,^6^,^7^ and permit no more than 2 meiotic crossovers per chromosome^8^,^9^. These features, and the absence of a centromere, substantially affect genome and karyotype evolution^10^,^11^,^12^,^13^,^14^,^15^,^16^ and make holocentric lineages useful model systems for studying various evolutionary phenomena, such as recombination rates and adaptability^17^, meiotic drive^18^,^19^, sex-chromosome evolution^20^,^21^,^22^ and homoploid hybridization^23^,^24^,^25^.

So far, holocentric chromosomes have been identified in plants, animals (see^1^,^2^,^26^ for detailed reviews) and in one ciliate species^27^. Among plants, they have been found in the green algal class Conjugatophyceae^28^ and in 2 families (Cyperaceae, Juncaceae) and 4 genera (*Myristica*, *Chionographis*, *Drosera* and *Custuta*) of flowering plants^2^. Among animals, holocentric chromosomes have so far been documented only in invertebrates, in which they have been confirmed in 10 orders of insects, 2 orders of centipedes, 6 orders of arachnids and 3 orders of Nematodes^1^,^26^. However, the known distribution of holocentric chromosomes across the tree of life is very likely an underestimate of their actual incidence, mainly because of methodical difficulties with their detection^2^,^29^,^30^.

Holocentric chromosomes can be identified based on the following criteria^2^,^26^,^30^: (i) a primary constriction is absent in mitotic metaphase; (ii) sister chromatids segregate parallel to each other in mitotic anaphase and, as a consequence, the anaphase plates appear as mirror images of one another; (iii) the kinetochore nearly spans the entire poleward surface of each chromatid in mitosis; and (iv) chromosomal fragments resulting from gamma or x-ray irradiation are normally inherited to daughter cells during subsequent cell divisions because they retain kinetochore activity. All of these criteria rely on microscopic observations of dividing cells and are reliable when applied to species with a small number of large chromosomes. However, these methods become unreliable in the case of small and numerous chromosomes, which often occurs with holocentric taxa^2^,^26^. In addition, karyological techniques are time-consuming and require a relatively high level of expertise. Collectively, present-day methods for the detection of holocentrism are not universally applicable and preclude high-throughput screening. However, the difference between monocentric and holocentric chromosomes in the inheritance of chromosomal fragments is a feature that can be, in principle, detected without the need for microscopic techniques.

Structural aberrations of monocentric chromosomes in dividing cells cause the unequal distribution of DNA in the daughter cells; therefore, a subsequently grown cell population is chimeric, containing cells that differ in their nuclear DNA content^31^. The increased nuclear DNA content variation in such a newly formed cell population is measurable using flow cytometry (FCM), as it causes an increase in the coefficient of variance (CV) of the G1 peaks in flow histograms^31^,^32^,^33^,^34^. After the induction of structural aberrations, the daughter cell population may also show a decrease in its mean nuclear DNA content, as has been shown in *Chrysanthemum*^35^ and *Rosa*^36^, both of which are monocentric (likely because of acentric chromosomal fragment loss).

In the present study, we aimed to address the possibility of microscope-independent detection of holocentric chromosomes in plants. Our approach is based on FCM measurements of nuclear DNA content in tissues formed after gamma irradiation of shoot meristems. Our strategy relies on the assumption that monocentrics will display more profound changes in the nuclear DNA contents of newly formed tissues than holocentrics because of their different chromosomal fragment inheritance, which should be detectable with FCM (see above). We evaluated the response to irradiation in a given species by comparing the FCM measurements of irradiated samples to non-irradiated controls.

## Materials and Methods

We examined representatives of 13 monocentric and 9 holocentric species (see Table 1). In addition, we included *Prionium serratum* (Thurniaceae), a species with an unknown chromosomal structure. *Prionium* is suspected to be holocentric, as it is closely related to the holocentric families Juncaceae and Cyperaceae and shares with them a very small genome and very low genomic GC content, in sharp contrast to their close monocentric relatives^37^. So far, Thurniaceae have never been karyologically studied.

To eliminate potential biases due to intraspecific variation in DNA content (cf.^38^), we preferred clonally propagated plants. For each species, all the samples (i.e., individual plant specimens) that were used for analysis had been cloned from a single individual, with the exception of *Pisum sativum* and *Silene nocturna,* whose samples were grown from seeds. We cultivated the samples in separate pots under standardized conditions in an experimental greenhouse (temperature, 20 °C; humidity, 90%; light period, 12 hours; light source, Philips GreenPower LED situated 50 cm above the growing table; spectrum, blue = 440 nm, deep red = 660 nm; PAR, 275 μmol m^−2 ^s^−1^). After 2 or 3 weeks, approximately two-thirds of the samples from each species were randomly chosen and irradiated with a 150 Gy dose of Cobalt-60 gamma irradiation (Bioster, Czech Republic), while the remaining samples were used as non-irradiated controls. We elected to irradiate two-thirds of the samples to compensate for potential irradiation-induced mortality.

As soon as the irradiated samples grew new tissues (leaves, or stems in *Eleocharis palustris*) from the irradiated meristems, typically after 2 or 3 weeks, we conducted FCM measurements. Only the newly grown tissues from irradiated samples and comparably young tissues from non-irradiated control samples were subjected to FCM. We performed FCM analyses on a CyFlow ML flow cytometer (Partec, Germany) that was equipped with a UV-LED diode excitation source. We used a DAPI fluorochrome, applying a 2-step sample preparation procedure^39^ and following the protocol of Šmarda *et al.*^40^. For each species, all of the irradiated and control samples were measured in a random order on a single day using internal standards (Supplementary Table S1).

**Figure 1:**
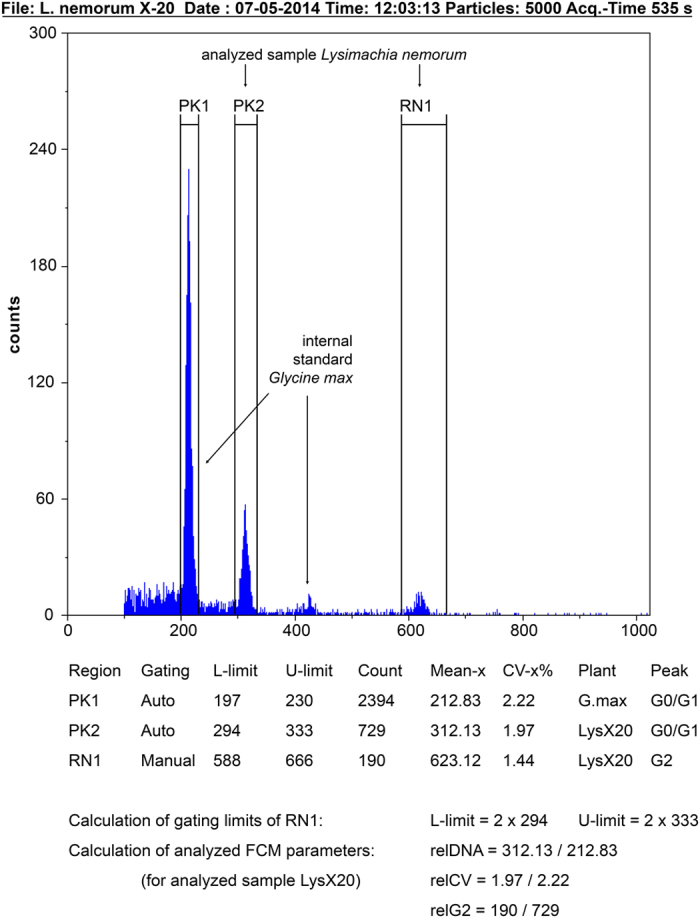
FCM histogram of irradiated sample X20 of *Lysimachia nemorum* with examples of gating limit calculations and with the cytometric parameters relDNA, relCV and relG2. X-axis, fluorescence intensity; y-axis, number of particles.

For each sample, we recorded 2 FCM parameters: (i) the relative nuclear DNA content (relDNA), calculated as the ratio of the mean of the G0/G1 peak of the sample to the mean of the G0/G1 peak of the internal standard, and (ii) the relative CV of the G0/G1 peak (relCV), calculated as the ratio of the CV of a sample G0/G1 peak to the CV of the corresponding G0/G1 peak of the internal standard (for an example calculation, see Fig. 1). As internal standardization is widely used for FCM detection of genome size^41^, we also analyzed the “intra-tissue” variation in nuclear DNA content using the CV as a proxy in the same way (i.e., in relation to the CV of the internal standard) to prevent potential biases, such as those caused by chopping intensity or instrument fluctuations. We later re-analyzed the raw FCM data (see the Results and Discussion for further explanation) to calculate another FCM parameter, namely (iii) the proportion of tetraploid nuclei in a sample (relG2), which we calculated as the number of G2 nuclei divided by the number of G0/G1 nuclei (for an example calculation, see Fig. 1). The position of the G2 peak was not always detected by automatic gating (FloMax software, Partec, Germany). Therefore, to standardize peak gating for the calculation of relG2, we used automated gating only for the G0/G1 peak, while the boundaries of the G2 peak were set to double the values of the lower and upper limits of the automated G0/G1 peak gating (for an example, see Fig. 1). To assess the response of a particular species to gamma irradiation with respect to any of the 3 FCM parameters, we compared the values of irradiated and control sample sets using Mann-Whitney U tests for each species and parameter.

Based on the comparison of irradiated and non-irradiated samples of each species, we also estimated the overall irradiation effect, which we calculated for relDNA as the mean relDNA value of irradiated samples/the mean relDNA value of control samples. We also made similar calculations for relCV and relG2. Finally, we compared these 3 overall irradiation effects between monocentric and holocentric species sets (using the Mann-Whitney U test) to assess whether monocentric and holocentric species differ in their response to gamma irradiation and whether any differences in relDNA, relCV or relG2 values are large enough to allow FCM to distinguish between holocentric and monocentric species.

## Results

In total, we cultivated 598 samples of 13 monocentric species, 9 holocentric species and 1 species with an unknown chromosomal structure (Supplementary Table S1). The overall sample mortality was 11.2%, leaving 531 samples for further FCM analyses. Although mortality was higher in irradiated samples, this effect did not differ between holocentric and monocentric species (p = 0.51, see Supplementary Figure S1 and Table S2). The appearance and overall physical condition of irradiated plants were worse than those of non-irradiated controls in both monocentric and holocentric species. Judging from visual inspection, gamma irradiation seemed to impact physical condition more severely in monocentric species than in holocentric species. However, this observation is a subjective impression, and the decrease in overall physical condition after gamma irradiation is unsuitable as a reliable parameter to distinguish between monocentrics and holocentrics.

**Figure 2:**
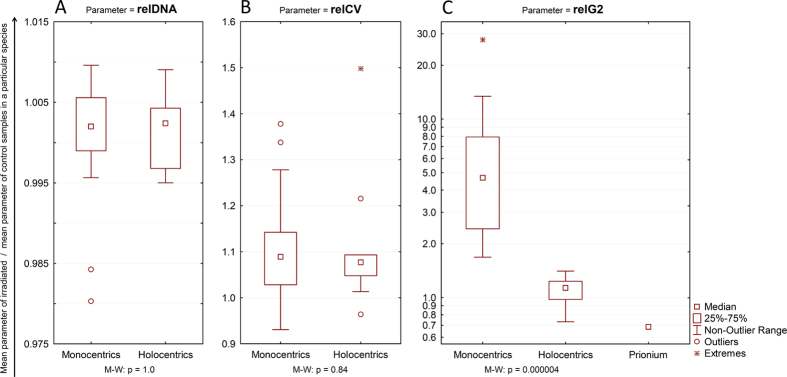
Differences between monocentrics and holocentrics with respect to the overall irradiation effects on nuclear DNA content (A), the CV of the G0/G1 peak (B) and the proportion of G2 nuclei plotted on a logarithmic scale (C). P-values (Mann-Whitney) are shown at the bottom.

Changes in relDNA induced by gamma irradiation varied from species to species. In some species, gamma irradiation caused an increase in relDNA, while other species responded with a decreased relDNA and still others displayed no change at all (Supplementary Figure S2). We did not detect any difference in the overall effect of irradiation on relDNA between monocentrics and holocentrics (Fig. 2A).

Intra-tissue variation in nuclear DNA content, measured as the relCVs of the G0/G1 peaks, increased in response to gamma irradiation in most species, but this increase typically did not reach significance (Supplementary Figure S3). As for relDNA, we did not detect any difference between monocentrics and holocentrics with respect to the overall irradiation effect on relCV (Fig. 2B).

Our prediction that radiation-induced changes in relDNA and relCV might distinguish between monocentrics and holocentrics using FCM was not confirmed by our experimental data. Hence, we developed an alternative hypothesis. We reasoned that if our subjective impression that irradiated holocentric plants were in better physical condition than irradiated monocentric plants (see above) were true, then perhaps the tissues of irradiated holocentrics contain more dividing cells than the tissues of irradiated monocentrics. The proportion of dividing cells can be measured using FCM as the proportion of the number of particles in the G2 peak of the sample (relG2; see Materials and Methods). Therefore, we returned to the raw FCM data and calculated this parameter for all of the samples. We expected that irradiation would be associated with lower relG2 values in both holocentrics and monocentrics and that the decrease might be more dramatic in monocentrics. Surprisingly, our findings were contrary to our expectations. The relG2 values in holocentric species either did not change or insignificantly increased after irradiation (Supplementary Figure S4). Monocentrics universally showed an increase in the proportion of G2 cells, and this increase was largely significant (in 12 of 13 species) or nearly significant (in *Lavandula angustifolia*) (Supplementary Figure S4). Moreover, the overall irradiation effect on relG2 in monocentrics was markedly stronger than in holocentrics, without any overlap between the 2 groups (Mann-Whitney p = 0.000004; Fig. 2C, Table 1, and Supplementary Table S2). The overall irradiation effect on relG2 in the species with an unknown chromosomal structure (*Prionium serratum*) was below the range of monocentric species, near the lower limit of holocentric species (Table 1, Fig. 2C).

The differences between monocentrics and holocentrics in the overall effects of irradiation on relDNA, relCV and relG2 remained the same when the median or the geometric mean (rather than the arithmetic mean; see Materials and Methods) was used to calculate the overall irradiation effect (see Supplementary Tables S1 and S2).

## Discussion

We combined gamma irradiation with FCM to address the possibility of making a microscope-independent distinction between chromosomal monocentrism and holocentrism in plants. Contrary to our expectations, we were unable to reliably distinguish between holocentrics and monocentrics based on changes in relDNA and relCV in response to gamma irradiation. However, relG2 was strongly elevated in irradiated monocentrics, while irradiated holocentrics were only negligibly affected (Fig. 2C). Our results suggest that a given plant species might be holocentric if it shows no significant increase in relG2 in irradiated samples relative to non-irradiated controls and/or if the overall irradiation effect for this parameter is up to approximately 1.5. *Prionium serratum*, a species with an unknown chromosomal structure but that was suspected to be holocentric^37^, met these conditions (Fig. 2C, Table 1). Therefore, it is likely that the monotypic genus *Prionium* is truly holocentric and thus that other members of Thurniaceae (a small sister family to the holocentric Cyperaceae and Juncaceae families) are good candidates for similar analyses. Another candidate might be the monotypic family Hydatellaceae, as recent evidence has suggested holocentrism in *Trithuria submersa*^42^.

The nearly total unresponsiveness of relG2 to gamma irradiation that we observed for holocentric plants is a surprising finding that is difficult to explain. It is known from cancer research that radiation therapy induces endopolyploidy in tumors^43^,^44^,^45^, and similar responses to ionizing irradiation have also been reported in *Arabidopsis*^46^,^47^, cucumber^48^ and pea^49^. Generally, plants appear to respond to DNA-damaging agents with cell cycle arrest, which leads to endopolyploidy^50^. To our knowledge, however, all previous studies of the endopolyploid response to DNA-damaging agents were performed with monocentric plants. Because holocentrics can easily cope with chromosomal fissions and fusions^3^,^4^,^5^,^6^,^7^, it is possible that holocentrics do not respond to DNA damage by cell cycle arrest to the same extent as monocentrics, and they thus show a much smaller increase in the proportion of G2 (endopolyploid) cells after gamma irradiation.

Whereas a single plant is typically sufficient for an FCM-based estimation of genome size in a particular species, the disadvantage of FCM-based detection of holocentrism is that it requires a statistically representative sample set. Moreover, unlike FCM-based genome size estimation, FCM-based detection of holocentrism requires longer sample cultivation and survival after irradiation, which could be problematic in species whose growth requires optimization. Conversely, larger and statistically representative sample sets for the analysis of newly formed leaves sufficiently prevent potential biases, including those due to variations in endopolyploidy, which is common in plant organs and tissues^51^.

We have shown that FCM may be a microscope-independent method by which to distinguish holocentrism from monocentrism in plants. It is very likely that other types of DNA-damaging irradiation or chemicals will serve similarly well or even better in combination with FCM. Unlike microscope-based karyological techniques, FCM does not require metaphase chromosomes and can easily be performed with interphase somatic cells. Although we found a parameter (relG2) that can reliably distinguish between monocentrics and holocentrics, it applies with certainty only to the species that we analyzed; therefore, further testing is required. However, we believe that FCM may now be used for high-throughput screening across plant phylogeny to search for candidate holocentric species.

## Additional Information

**How to cite this article**: Zedek, F. *et al.* Flow cytometry may allow microscope-independent detection of holocentric chromosomes in plants. *Sci. Rep.* **6**, 27161; doi: 10.1038/srep27161 (2016).

## Supplementary Material

Supplementary figures S1-S4

Supplementary tables S1 and S2

## Figures and Tables

**Table 1:** Overall irradiation effects on mortality, nuclear DNA content, the coefficient of variance of the G0/G1 peak and the proportion of G2 nuclei, based on comparisons of irradiated and control samples.

Species	Family	Chromosomes	Overall irradiation effects on:			
			Mortality	Nuclear DNA content	G0/G1 peak CV	G2 nuclei proportion
*Asplenium bulbiferum*	Aspleniaceae	mono	0.92	1.00	1.00	1.68
*Begonia bowerae*	Begoniaceae	mono	1.08	1.00	1.28	6.03
*Carex grayi*	Cyperaceae	holo	1.00	1.00	1.05	1.13
*Carex humilis*	Cyperaceae	holo	1.33	1.03	1.11	1.13
*Carex pilulifera*	Cyperaceae	holo	1.17	1.00	1.09	1.41
*Cymbalaria muralis*	Plantaginaceae	mono	1.25	1.00	1.38	7.93
*Drosera capensis*	Droseraceae	holo	1.13	1.00	1.05	0.86
*Drosera scorpioides*	Droseraceae	holo	1.00	1.01	1.22	1.12
*Eleocharis palustris*	Cyperaceae	holo	1.00	1.01	1.50	0.73
*Euonymus japonicus*	Celastraceae	mono	1.05	0.98	0.99	8.12
*Isolepis prolifera*	Cyperaceae	holo	1.30	1.00	1.08	1.22
*Kalanchoë delagoensis*	Crassulaceae	mono	1.13	0.98	1.34	2.43
*Lavandula angustifolia*	Lamiaceae	mono	1.06	1.01	1.03	2.47
*Luzula sylvatica*	Juncaceae	holo	1.00	1.00	0.96	1.23
*Lysimachia nemorum*	Primulaceae	mono	1.00	1.00	0.93	4.90
*Peperomia glabella*	Piperaceae	mono	1.20	1.01	1.09	2.23
*Pisum sativum*	Fabaceae	mono	0.78	1.01	1.12	13.40
*Plectranthus amboinicus*	Lamiaceae	mono	1.00	1.01	1.05	2.04
*Prionium serratum*	Thurniaceae	unknown	1.00	1.00	1.03	0.69
*Scirpus cernuus*	Cyperaceae	holo	1.05	1.00	1.01	1.26
*Sedum spurium*	Crassulaceae	mono	1.06	1.00	1.11	3.80
*Senecio articulatus*	Asteraceae	mono	1.00	1.00	1.08	27.78
*Silene nocturna*	Caryophyllaceae	mono	1.00	1.01	1.14	4.68

Mono = monocentric chromosomes; holo = holocentric chromosomes; CV = coefficient of variance. The overall irradiation effect on mortality was calculated as the mean relMort of irradiated/mean relMort of control samples. Similar calculations were used for nuclear DNA content, G0/G1 peak CV, and for G2 nuclei proportion with relDNA, relCV, and relG2, respectively, where relMort = number of dead samples/total number of samples, relDNA = mean of the sample G0/G1 peak/mean of the G0/G1 peak of the internal standard, relCV = CV of a sample G0/G1 peak/CV of the G0/G1 peak of the internal standard, relG2 = number of G2 nuclei/number of G0/G1 nuclei (for primary data, see Supplementary Table S1).
