# Corrigendum: Flow cytometry may allow microscope-independent detection of holocentric chromosomes in plants

**DOI:** 10.1038/srep46869

**Published:** 2017-06-30

**Authors:** František Zedek, Pavel Veselý, Lucie Horová, Petr Bureš

Scientific Reports 6: Article number: 2716110.1038/srep27161; published online: 06 03 2016; updated: 06 30 2017

This Article contains typographical errors in the Acknowledgements section.

“We would like to thank to Adéla Jirků, Luboš Maťaš and Jan Hladík from Bioster Company for all their efforts and assistance with the gamma irradiation of our samples”.

should read:

“We would like to thank to Adéla Jirků, Luboš Maťaš and Josef Hladík from Bioster Company for all their efforts and assistance with the gamma irradiation of our samples”.

