# Supplementary material for: Flow cytometry may allow microscope-independent detection of holocentric chromosomes in plants
Source: Sci Rep. 2016 Jun 3;6:27161. doi: 10.1038/srep27161 (PMC4891681; doi:10.1038/srep27161)
Supplement: Supplementary figures S1-S4 [file srep27161-s1.pdf]

Flow cytometry may allow microscope-independent detection of holocentric chromosomes in plants

František Zedek, Pavel Veselý, Lucie Horová & Petr Bureš

Supplementary figures S1-S4

Figure S1. Overall irradiation effect on sample mortality: comparison between monocentrics and holocentrics

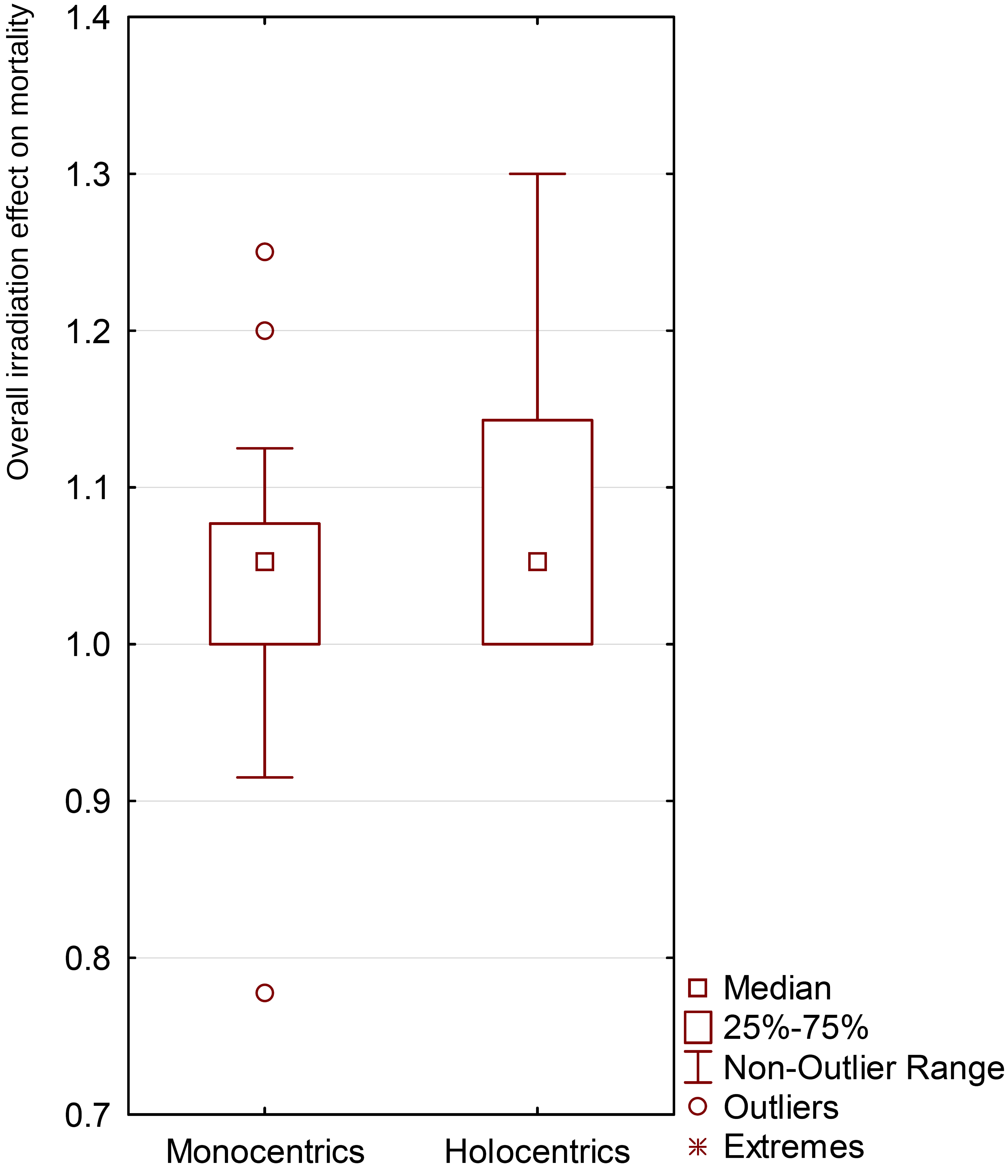

The overall irradiation effect on mortality was calculated as 1 / survival. Survival was calculated as % of surviving irradiated samples / % of surviving control samples for a particular species. Monocentrics and holocentrics did not differ in their radiation-induced mortality (Mann-Whitney, $p = 0.51$). Actual values and calculations are given in Supplementary Table S2.

Figure S2. Change of relDNA after irradiation in:

monocentrics   holocentrics

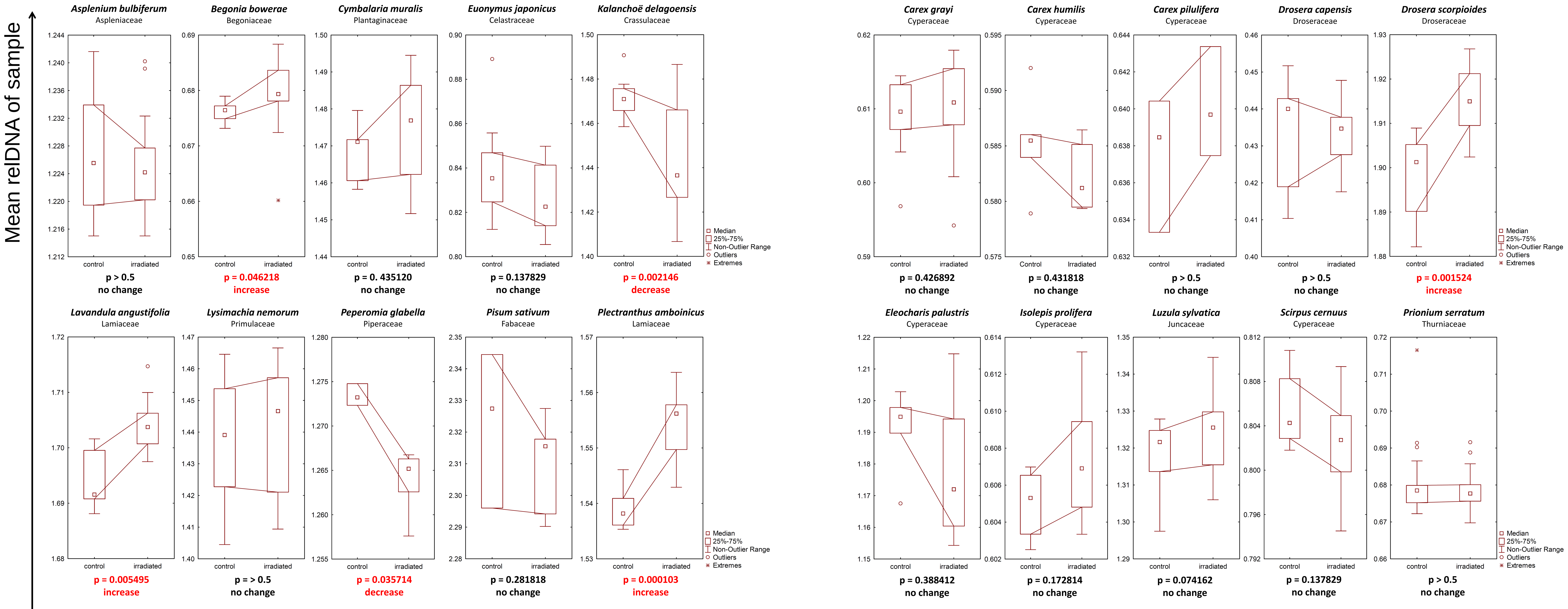

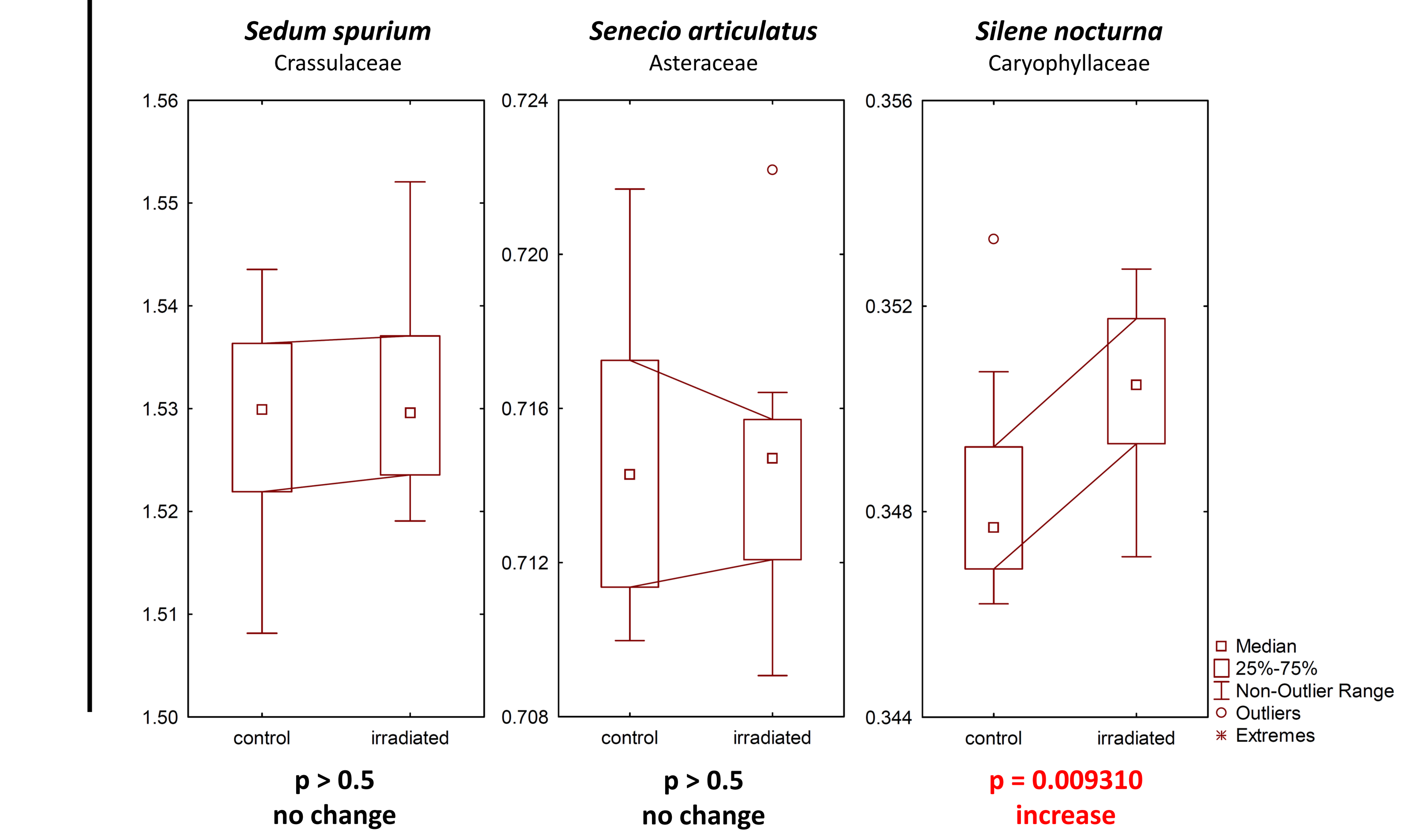

Each graph is a box-plot comparison of the relDNA values of control samples and the relDNA values of irradiated samples for a given species. Significant differences were tested with the Mann-Whitney U test. Each relDNA value (relative nuclear DNA content) is calculated as the ratio of the mean of the G0/G1 peak of the sample to the mean of the G0/G1 peak of the internal standard for a given FCM measurement (see Figure 1 for a calculation example and Supplementary Table S1 for actual values).

Figure S3. Increase in relCV after irradiation in:

monocentrics

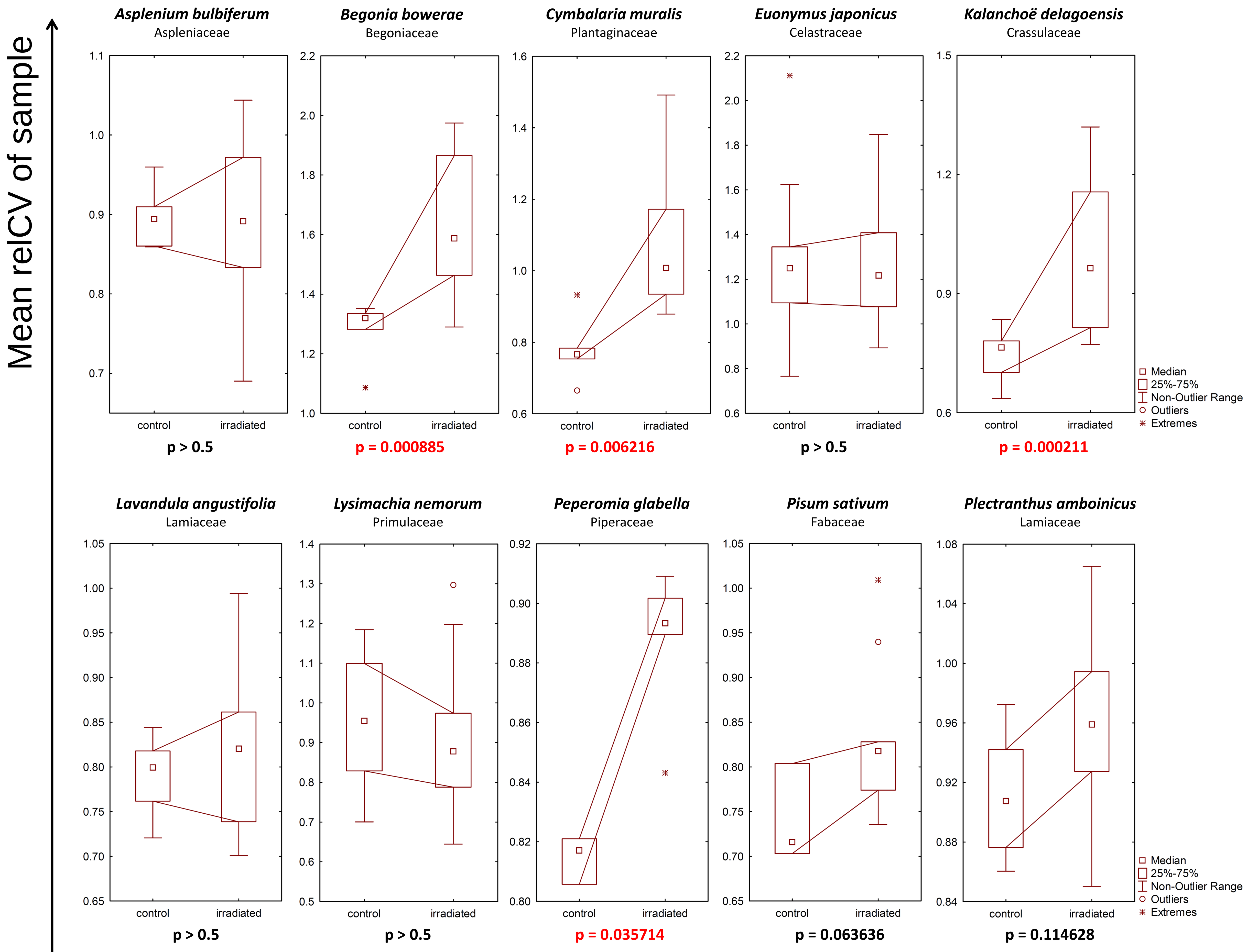

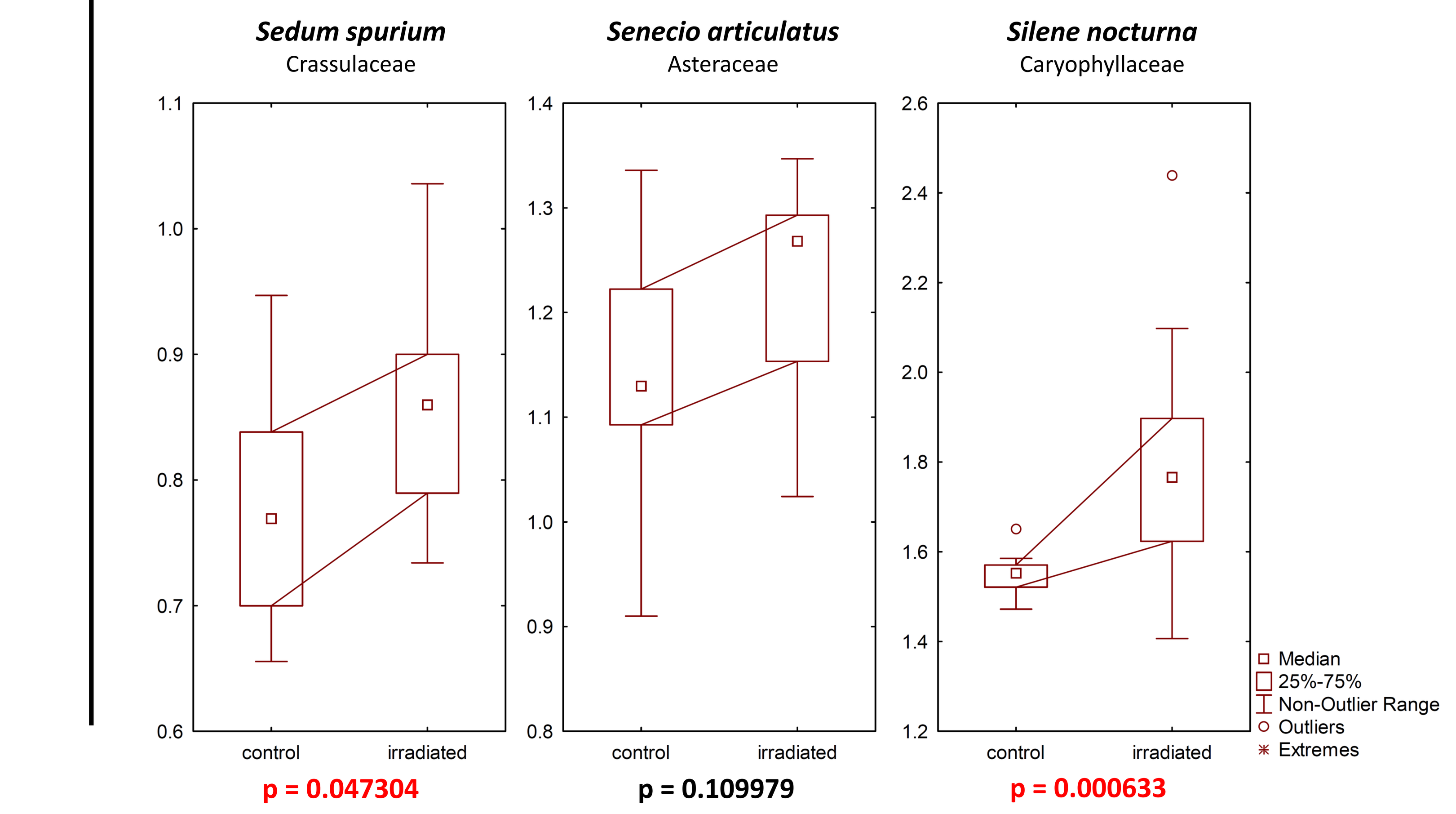

holocentrics

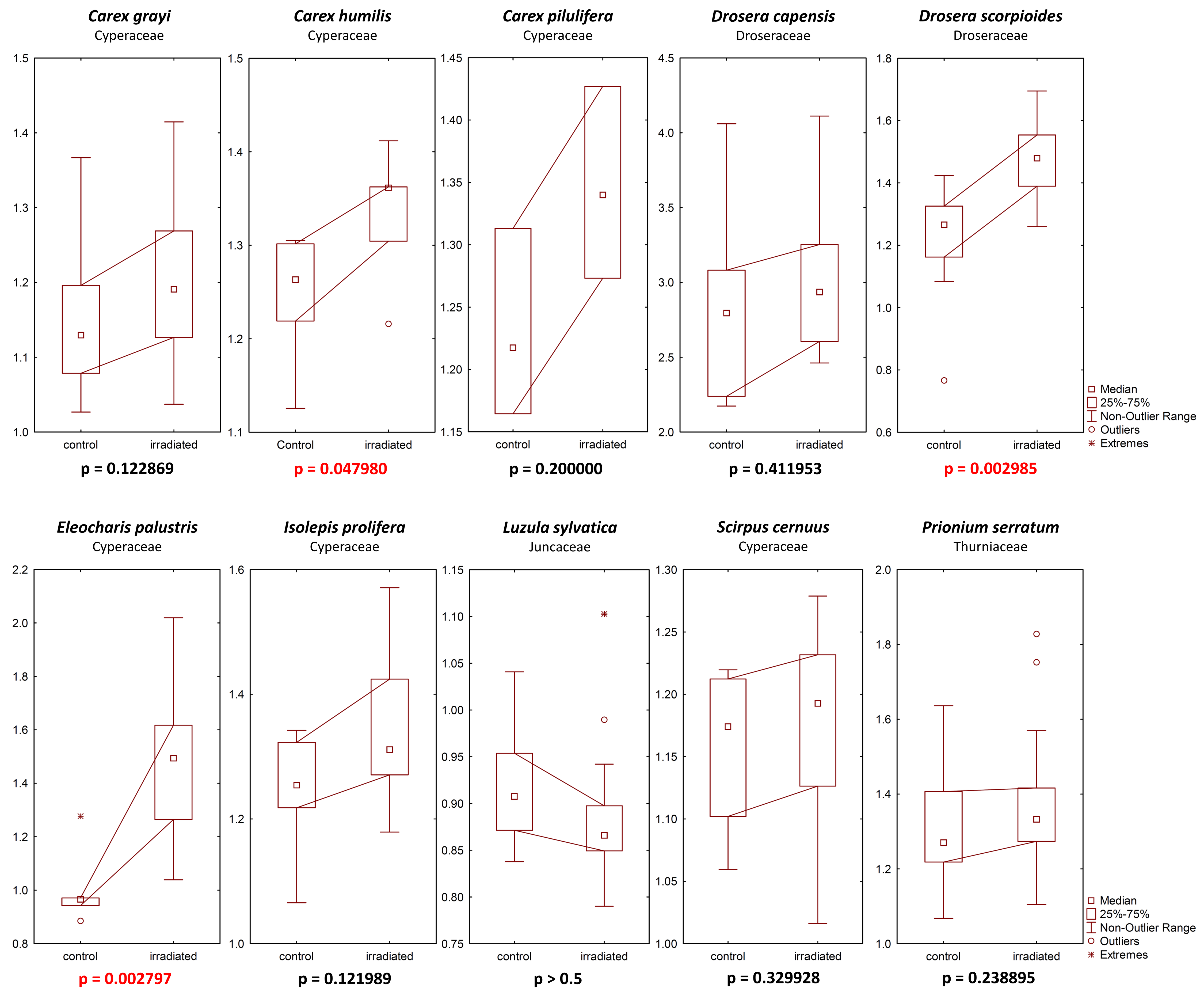

Each graph is a box-plot comparison of the relCV values of control samples and the relCV values of irradiated samples for a given species. Significant differences were tested with the Mann-Whitney U test. Each relCV value (relative coefficient of variation) is calculated as the ratio of the CV(%) of the G0/G1 peak of the sample to the CV(%) of the G0/G1 peak of the internal standard for a given FCM measurement (see Figure 1 for a calculation example and Supplementary Table S1 for actual values).

Figure S4. Increase in relG2 after irradiation in:

monocentrics

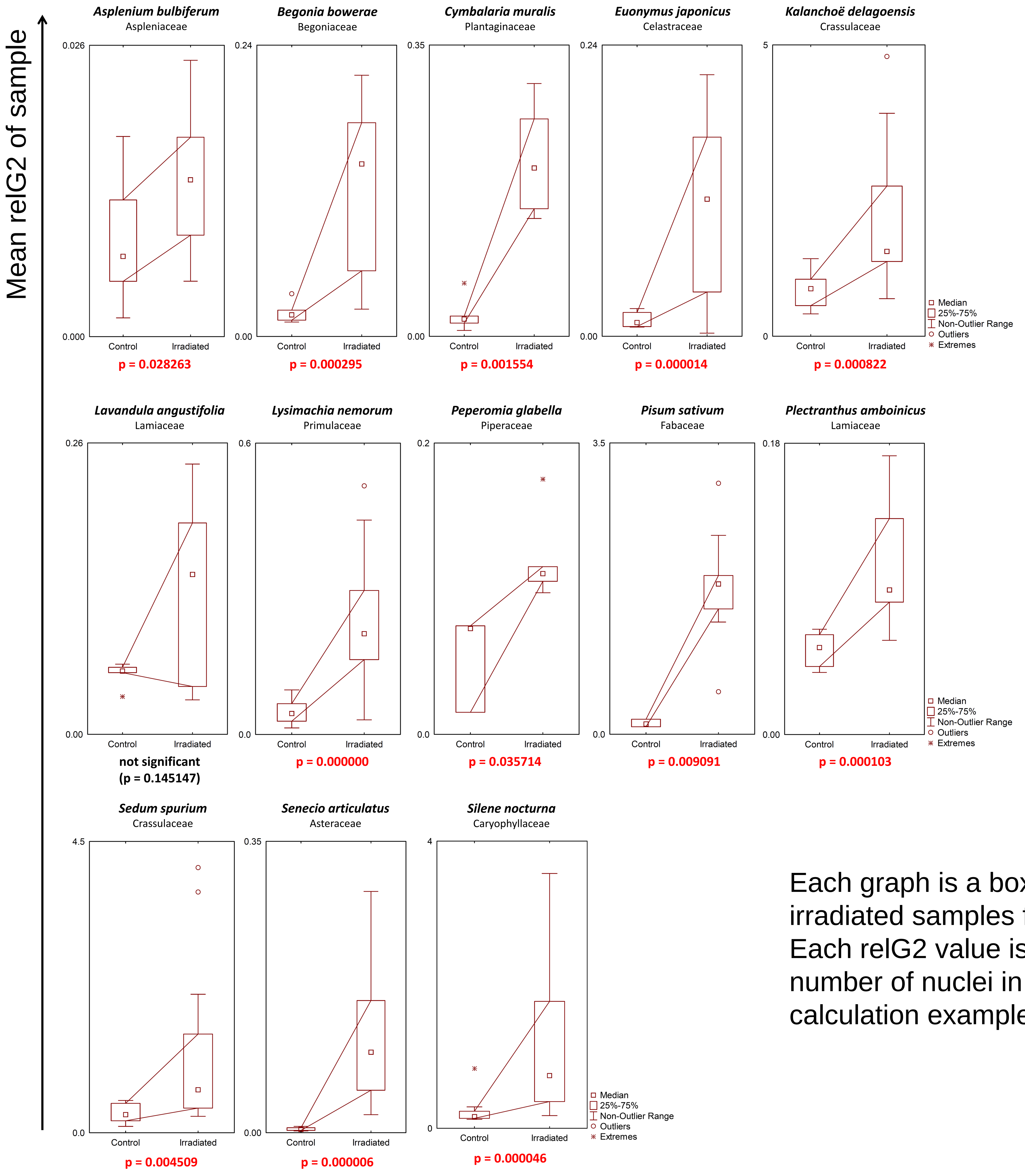

holocentrics

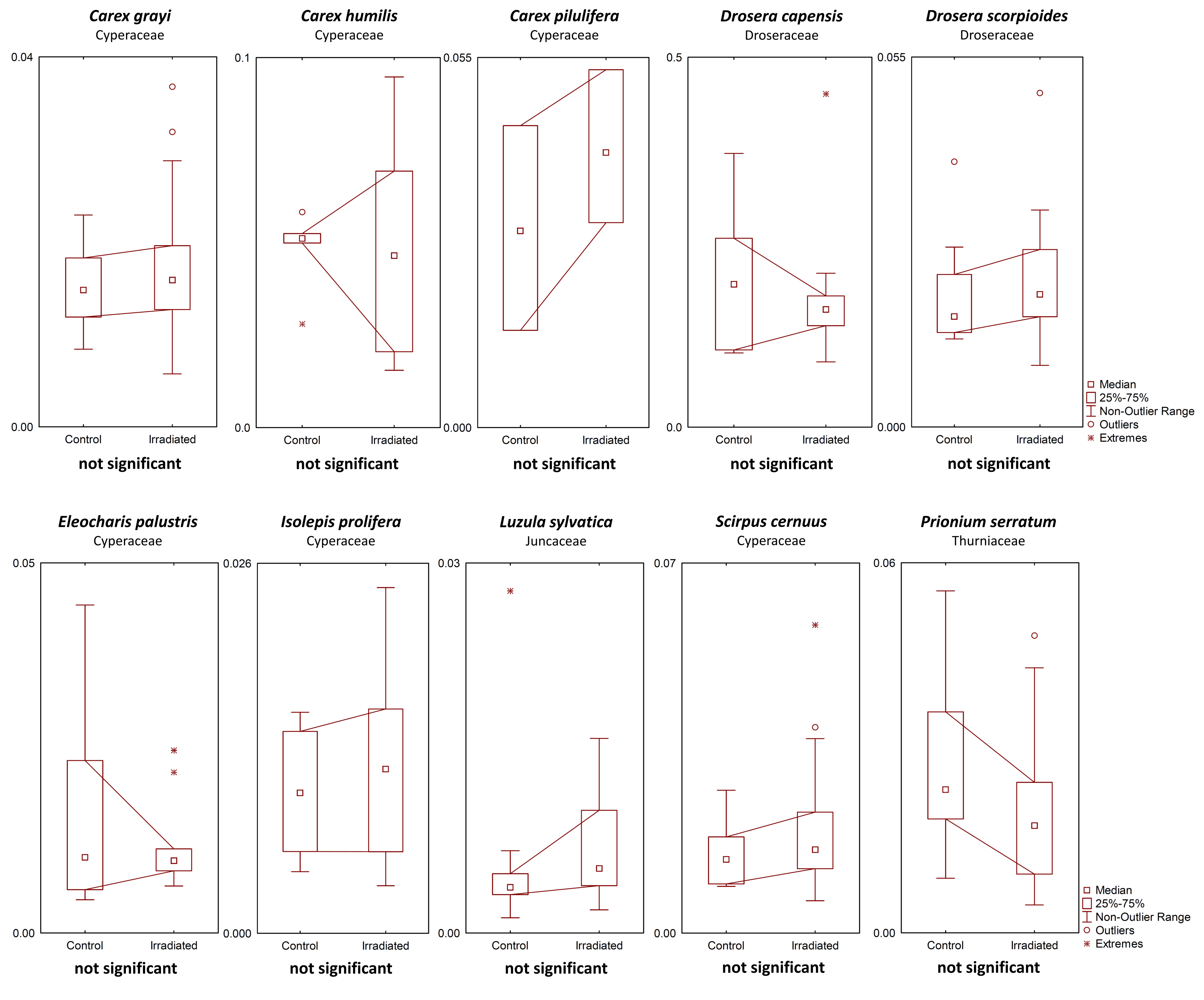

Each graph is a box-plot comparison of the relG2 values of control samples and the relG2 values of irradiated samples for a given species. Significant differences were tested with the Mann-Whitney U test. Each relG2 value is calculated as the ratio of the number of nuclei in the G2 peak of the sample to the number of nuclei in the G0/G1 peak of the sample for a given FCM measurement (see Figure 1 for a calculation example and Supplementary Table S1 for actual values).
